# Ring-shaped Racetrack memory based on spin orbit torque driven chiral domain wall motions

**DOI:** 10.1038/srep35062

**Published:** 2016-10-11

**Authors:** Yue Zhang, Xueying Zhang, Jingtong Hu, Jiang Nan, Zhenyi Zheng, Zhizhong Zhang, Youguang Zhang, Nicolas Vernier, Dafine Ravelosona, Weisheng Zhao

**Affiliations:** 1 Fert Beijing Institute, Beihang University, Beijing, China; 2 School of Electronic and Information Engineering, Beihang University, Beijing, China; 3 Institut d’Electronique Fondamentale (IEF), Univ. Paris-Sud, CNRS, Orsay, France; 4 School of Electrical and Computer Engineering, Oklahoma State University, USA

## Abstract

Racetrack memory (RM) has sparked enormous interest thanks to its outstanding potential for low-power, high-density and high-speed data storage. However, since it requires bi-directional domain wall (DW) shifting process for outputting data, the mainstream stripe-shaped concept certainly suffers from the data overflow issue. This geometrical restriction leads to increasing complexity of peripheral circuits or programming as well as undesirable reliability issue. In this work, we propose and study ring-shaped RM, which is based on an alternative mechanism, spin orbit torque (SOT) driven chiral DW motions. Micromagnetic simulations have been carried out to validate its functionality and exhibit its performance advantages. The current flowing through the heavy metal instead of ferromagnetic layer realizes the “end to end” circulation of storage data, which remains all the data in the device even if they are shifted. It blazes a promising path for application of RM in practical memory and logic.

Employing magnetic domains as information carriers is currently one of the most attractive orientations of spintronics, owing to its non-volatility, extremely small size as well as controllability. Domain walls (DWs) are used for describing vividly the transition regions between different magnetizations^1^,^2^. Although magnetic field driven DW motion has been observed for a long time and is relatively easy to understand, unidirectional current-induced DW motions provide us a feasible way for truly achieving low-power, high-density and high-speed data storage^3^,^4^. Racetrack memory (RM) is an important concept based on this phenomenon, which keeps drawing great interest from both academics and industries^5^,^6^,^7^,^8^.

For a long time, current-induced DW motions only refer to spin transfer torque (STT) mechanism, in which the direction of DW motions should be the same with that of the electron flow, i.e. against that of the applied charge current. Recently, DW motions in the charge current direction have been observed. Although there is no consensus for its concrete origin, spin orbit torque (SOT), instead of STT, is considered to play a dominate role. Unlike the current injected directly into ferromagnetic layer in the case of STT, a charge current passing through a nonmagnetic conductive layer with strong spin orbit coupling (e.g. heavy metals) could induce magnetization switching in the adjacent magnetic layer. In order to understand and apply this emerging phenomenon, Rashba effect, spin Hall Effect (SHE) as well as Dzyaloshinskii-Moriya interaction (DMI) have been put forward and investigated intensely^9^,^10^,^11^,^12^,^13^,^14^,^15^,^16^. Compared with conventional STT, SOT provides more efficient DW motions, which require smaller shift current and perform higher velocity. Moreover, the charge current flows through the heavy metal layer whose resistivity might be lower than that of ferromagnetic layer, which allows less voltage supply and less power consumption.

The conventional structure of RM is stripe-shaped (see Fig. 1a), where the two ends of nanowire cannot be connected^17^. It suffers from some formidable obstacles hindering its practical application for future electronics. Among them, the data overflow issue is normally considered as its Achilles’ heel: as the data are serially stored in RM, the operations on an individual bit certainly stimulate synchronous motions of the others; in the stripe-shaped case, this would lead to a part of data moving out of the device. Some attempts might solve this problem, for example, adding peripheral registers for storing the overflowing data, or extending the nanowire for avoiding the overflow^18^. However, these remedial methods unavoidably result in performance degradation in aspects of density, speed and power consumption^19^ proposed a ring structure with DW ratchets, which could be utilized for addressing the data overflow issue without any additional overhead. However, it still relies on the manipulation of magnetic field and is difficult to realize large capacity and high density. In this work, we develop and study a ring-shaped RM based on SOT driven chiral DW motions. As shown in Fig. 1c, spin accumulation in ferromagnetic layer arises from an in-plane current applied to the underneath heavy-metal layer^20^,^21^,^22^,^23^,^24^,^25^. This structure can achieve “end to end” data storage, allowing the data remaining in the nanowire even in the shifting process. Micromagnetic simulations have been carried out to confirm its functionality. A series of the crucial problems concerning its feasibility are discussed and optimized. This study will promote the development of RM towards commercialization and inspire more innovative memory and logic concepts.

## Results

### Ring-shaped RM structure and material configurations

**Figure 1:**
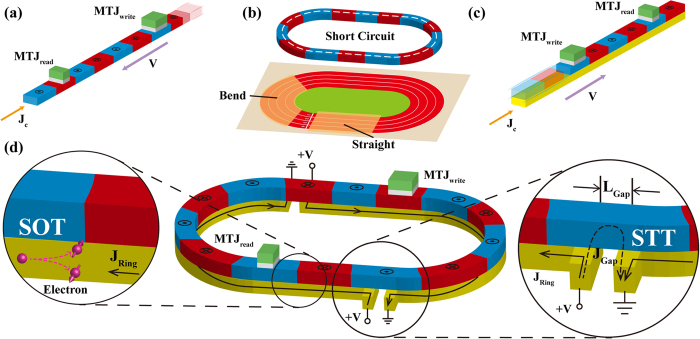
(**a**) Conventional spin transfer torque (STT) driven racetrack memory (RM), in which current flows through the ferromagnetic (data storage) layer. (**b**) Real racetrack shape leads to short-circuit issue for STT driven RM. (**c**) Spin orbit torque (SOT) driven RM, in which current is injected in the heavy metal layer underneath the data storage layer. (**d**) Ring-shaped SOT driven RM schematic. Domain walls (DWs) can be nucleated and detected by magnetic tunnel junctions (MTJ_write_ and MTJ_read_). Two gaps are introduced to avoid the short-circuit issue. Current flows through heavy metal layer and leaks partially through the ferromagnetic layer above the gap (see inset). The leakage current could also induce DW motions, which guarantees the continuation of data transfer.

Figure 1d shows the schematic of the ring-shaped RM. From the functional point of view, it is composed of three parts: write head for DW nucleation, read head for magnetization detection and nanowire for DW motions. The write and read heads can be constructed by magnetic tunnel junctions (i.e. MTJ_write_ and MTJ_read_), as same as those for the conventional RM^26^,^27^. Particularly for the magnetic nanowire, the magnetic layer (e.g. CoFeB) is entirely closed while the adjacent heavy metal layer (e.g. Pt, W, Ta, Hf) has tiny gaps for avoiding short-circuit (see Fig. 1b). When the voltages are given on these gaps, due to the small resistance of heavy metal, the current mainly flows through the heavy metal layer, which thus induces SOT driven chiral DW motions in the parts apart from the gap. On the other hand, a leakage current can still flow through the magnetic layer over the gap part (see inset of Fig. 1d), whose value is related to the size of the gap. If the value is high enough, STT can be exerted in the gap part. The influence of the gap on the whole structure will be investigated in the following discussion section.

It is noteworthy that the direction of chiral DW motions driven by SOT can be either along or against the charge current direction in heavy metal layer. This depends on the sign of spin hall angle and DW chirality of given multilayer system, for example, DWs move along the direction of current flow in W/CoFeB/MgO or Pt/CoFeB/MgO sandwich system, while in Ta/CoFeB/MgO or Hf/CoFeB/MgO, the case is reverse^19^,^20^,^21^,^22^,^23^,^24^,^25^. Here the oxide layer is used for enhancing the perpendicular magnetic anisotropy (PMA)^28^. As the direction of STT driven DW motions is always against the current direction, we choose Pt/CoFeB/MgO as the material configuration in order to shift the data circularly and continuously in the ring structure. In addition, Pt has a relatively low resistivity among these heavy metals, which is also much lower than that of ferromagnetic materials, such as CoFeB. Although recent progresses show that β-phase Ta and W can provide higher spin hall angle^25^, their high resistivity are unfavorable to the ring-shaped design.

### The “Straight” and the “Bend”

From the point of view of DW trajectory, the ring-shaped RM can be divided into two parts: the “Straight” and the “Bend”. The behaviors of DWs in these two parts are investigated via micromagnetic simulations with Mumax by introducing SOT and DMI into the Landau-Lifshitz-Gilbert (LLG) equation^29^ (See Methods for the parameters details).

**Figure 2:**
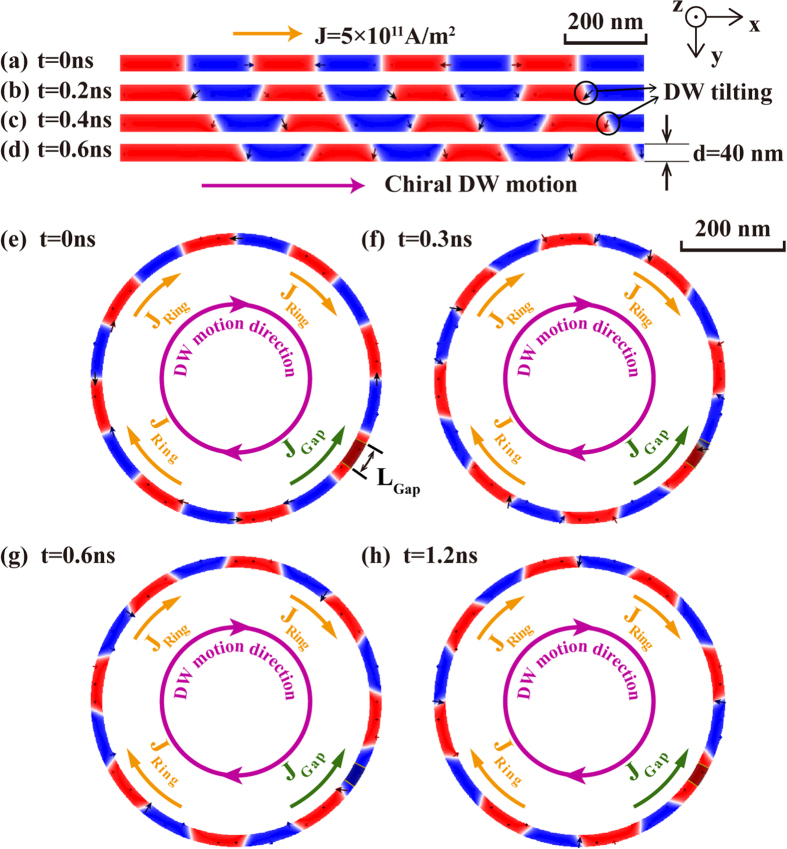
The top-view of the SOT driven chiral DW motions in the “Straight” and “Bend”. Red represents + z magnetization, blue represents –z magnetization. (**a–d**) The “Straight”: 40 nm-wide and 1.28 μm-long Pt(2 nm)/CoFeB(0.6 nm)/MgO. DW motion velocity can reach 400 m/s with a current of 2 × 10^11^ A/m^2^. DW tilting occurs which agrees with experimental results. (**e–h**) The “Bend”: external diameter *D*_*ex*_ is 1.024 μm, width d is 40 nm. The gap length of the heavy metal layer *L*_*gap*_ is 100 nm. Current injected into the heavy metal layer for SOT part *J*_*Ring*_ is 3 × 10^11^ A/m^2^ and leakage current in the gap part inducing STT *J*_*Gap*_ is 1.4 × 10^12^ A/m^2^. The different directions of current for these two parts guarantee the uni-direction of DW motions.

Firstly, Fig. 2a–d demonstrate the chiral DW motions in the “Straight”: a 40-nm-wide 1.28-μm-long nanowire of Pt(2 nm)/CoFeB(0.6 nm)/MgO (see also Supplementary Movie 1). DW motion velocity can reach 400 m/s with a current of 2 × 10^11^ A/m^2^. The motion velocity increases with the magnitude of injected current and approaches the saturation when the current exceeds 3 × 10^11^ A/m^2^. Secondly, Fig. 2e–h show the chiral DW motions in the “Bend” (see also Supplementary Movie 2). The geometry of magnetic layer is designed to be a circle whose external diameter is 1.024 μm, width is 40 nm. The gap length of the heavy metal layer is fixed to 100 nm. The material parameters are the same with those of the “Straight” given above. *J*_*Gap*_ and *J*_*Ring*_ are the current densities through the gap and ring parts which are 1.4 × 10^12^ A/m^2^ and 3 × 10^11^ A/m^2^. With the effects of the STT on the gap part and the SOT on the other part, DWs move along the ring synchronously.

**Figure 3:**
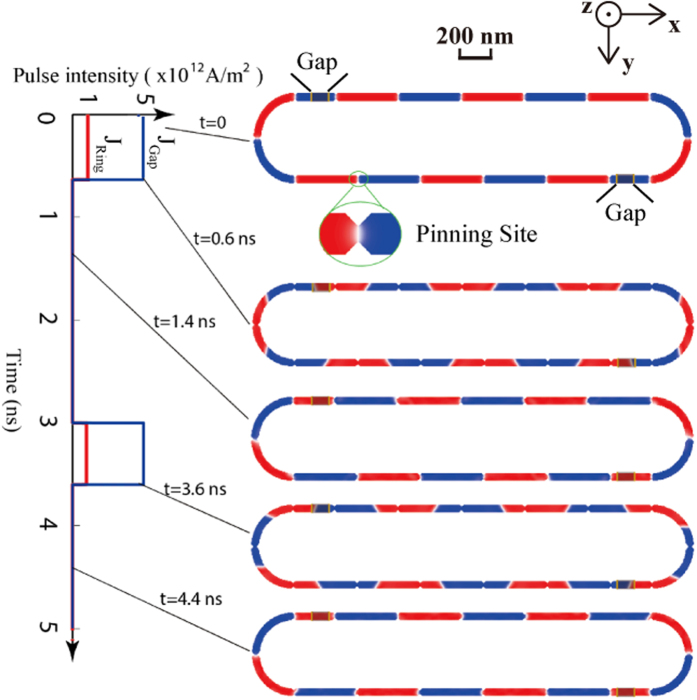
16-bit ring-shaped RM schematic. Two gaps and symmetric pinning sites (see inset) are used for ensuring the functionality and stability of the RM. *J*_*Gap*_ and *J*_*Ring*_ are 5 × 10^12^ A/m^2^ and 1 × 10^12^ A/m^2^. The length of gap is 100 nm each. 0.6 ns current pulses are sufficient for DW motions due to the inertia.

Compared with the circular structure, a more complex and concrete structure combining the “Straight” and the “Bend” (see Fig. 1b) is investigated to demonstrate the feasibility and reliability of our proposed ring-shaped RM. Figure 3 shows the complete process of chiral DW motions in the 16-bit ring-shaped RM (see also Supplementary Movie 3). Two gaps are inserted to keep the device geometrical symmetry. The length of gap is 100 nm each and the entire device length is 5.2 μm. Here, adding the pinning sites is an important mean to optimize the controllability of DW shifting. Note that the DMI stabilized SOT induced DWs are tilted and the tilting directions of the neighboring DWs alternate. In order to make the equal effect on DWs with different configurations, the pinning sites should be symmetry on both sides of the racetrack (see Supplementary Information). According to the resistivity of Pt and CoFeB^12^,^13^,^25^, *J*_*Gap*_ and *J*_*Ring*_ are 5 × 10^12^ A/m^2^ and 1 × 10^12^ A/m^2^ respectively, which are larger than those in Fig. 2, aiming to overcome the additional forces from the pinning sites. From the results, due to the DW motion inertia, the DWs can move synchronously and fluently with a 0.6-ns current pulse.

## Discussion

Stable data storage and data transmission are the most crucial for a memory application. Correspondingly, as the information carrier, DWs in RM should be studied in term of stability. Here, the status and the velocity of DWs will be discussed.

**Figure 4:**
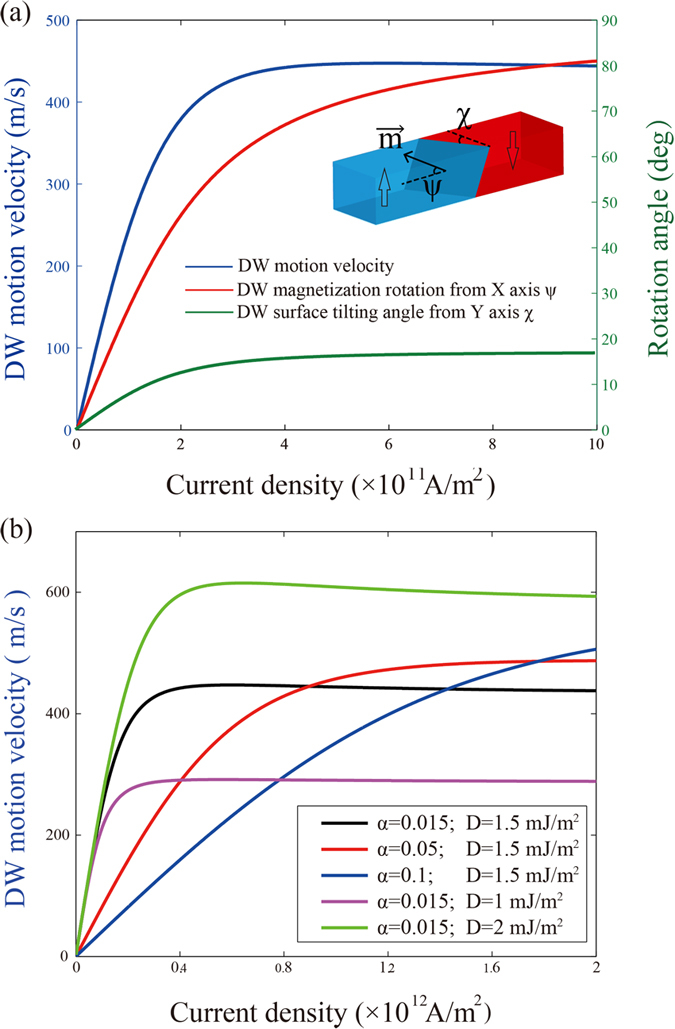
(**a**) The dependence of the DW motion velocity (blue), the DW magnetization rotation angle (red) and the DW tilting angle (green) versus the applied current density. Due to the DW magnetization rotation and tilting, the velocity will be saturated with a high current density. (**b**) The dependence of the DW motion velocity versus the damping and DMI constants. Lower damping and stronger DMI allows higher velocity for low current densities.

From the micromagnetic simulation results shown in Fig. 2, DWs are found to tilt away from the transverse axis, which agrees well with the experimental observations^30^. This DW tilting is the result of a balance between magnetic energy which is proportional to the DW surface and the energy related to the effective field induced by spin orbit interaction^31^. When the applied current increases, the effective field rotates the magnetization of DW away from longitude direction where the torque induced by SOT reaches its maximum. Taking DW tilting angle *χ* into account, the dynamics of SOT driven DW motions can be described by one-dimension (1-D) model as follows,


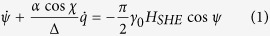



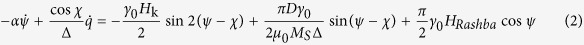



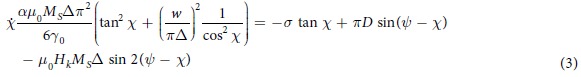


where *q* denotes the center position of DW, *ψ* is the magnetization direction of DW in x-y plane, Δ is the DW width, *H*_*k*_ is the anisotropy field, *α* is the Gilbert damping, *w* is the racetrack width, *σ* is the wall energy per unit area, the torques produced by SOT are added into the 1-D model equations via the effective fields, *H*_*SHE*_ and *H*_Rashba_ (See Methods for parameters details). As shown in Fig. 4a, with the increase of the current density, the DW magnetization rotation angle *ψ* and DW tilting angle will approach their saturations, 80 degrees and 18 degrees respectively. This will also lead to a saturation of DW velocity.

Indeed, the stability of DWs in the curved trajectory is a crucial point for guaranteeing the functionality of the ring-shaped RM, especially with respect to different DW configurations (up-down and down-up). By changing the ratio of nanowire width and curvature radius, we find that the velocities of different DWs have not an obvious difference (see Supplementary Information and Supplementary Movie 4). From the theoretical point of view, as the charge current in the heavy metal layer flows with a curved trajectory, the polarization of induced spin current is always vertical to the applied current, which is identical with the stripe-shaped case. Moreover, the tilting angles of different DW configurations are also steady in the curved trajectory. This can be explained by the origin of the DW tilting, i.e. the system will be steady in a configuration with the minimized energy. Although the curved shape tends to change the tilting angle during DW shifting process, this would also enlarge the DW surface, which runs in opposite direction for achieving the lowest energy^31^. On the other hand, the non-uniform current distribution and pinning factors, e.g. geometrical constrictions, inhomogeneous exchange stiffness and anisotropy, might occur in the proposed RM, which could lead to the velocity difference of different DW configurations or the other unexpected reliability issues. However, these can be mitigated by reducing the nanowire width, increasing the applied current or improving the technical process (see Supplementary Information). Therefore, the DW status will be stable in both the “Straight” and the “Bend”.

The DWs should also move with comparative speeds in ring part and gap part. The tiny velocity difference between these parts can be compensated by adjudging the ratio of the lengths of ring and gap. In the steady state, 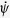, 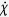 = 0 and 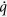 represents the velocity of SOT driven DW motions. From the 1-D model calculations, the velocity is related to the damping and DMI constants (see Fig. 4b). Higher damping and lower DMI constant lead to slower DW motion for relatively low current densities. Due to the high efficiency of SOT effect, this velocity is much higher than that of STT driven DW motions, which has also been proved by theoretical and experimental results^19^,^20^,^21^,^22^,^23^,^24^,^25^. However, in the simulations that we performed, we didn’t observe the large difference of DW motion velocity in different parts of the ring-shaped RM. This can be explained as follows. Firstly, the roughness is neglected in our simulations and the ferromagnetic material that we applied, CoFeB, has a relatively small damping constant. This allows the DWs to continue moving over a distance under their own inertia^32^,^33^. Secondly, since the gap is too small compared to the whole ring, the velocity will not decease enormously. On the other hand, if the current flowing over the gap is high enough to induce STT, this torque will also provide a motive force for DW motions along their original orientation. For the real device with significant roughness, the impact of STT becomes more significant, because it can ensure the continuous DW motions in the gap part. This improves the feasibility of the proposed ring-shaped RM.

**Figure 5:**
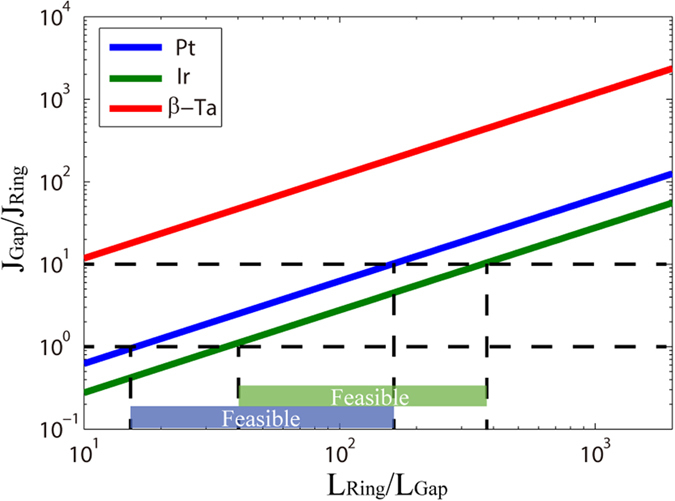
The dependence of the current density ratio of gap and ring parts versus the length ratio of gap and ring parts for different heavy metals. Assuming a typical range of current density ratio (e.g. 1~10), a feasible range of length ratio can be obtained.

From the analyses above, we find that the gap size is another important factor for realizing ring-shaped RM. Since the same voltage is charged on the gap and the ring, the current densities through the ring (heavy metal) *J*_*Ring*_ and the gap (ferromagnet) *J*_*Gap*_ should meet the following condition:


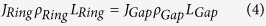


where *ρ*_*Ring*_ and *ρ*_Gap_are the resistivity of the heavy metal layer and the ferromagnetic layer, *L*_*Ring*_ and *L*_*Gap*_ are the lengths of the ring (without gap) and the gap. Figure 5 shows the dependence of current density on length for the two parts. Assuming the STT required current density is always 1~10 times of the SOT required current density, a feasible range can be extracted: if the gap is extremely small, the current passing over the gap could be too large to destroy the device; if the gap is considerably large, the DW velocities are so different between the ring and gap that multiple DWs perhaps sinks into the gap part, affecting the storage functionality and leading to reliability issue. Moreover, we compare various heavy metals with different resistivity. For example, the length ratio of 20~200 is appropriate for Pt; for Ir, whose resistivity is lower, the length ratio can be increased. This also proves that high resistivity (e.g. β-Ta) is not suitable for realizing this structure.

**Figure 6:**
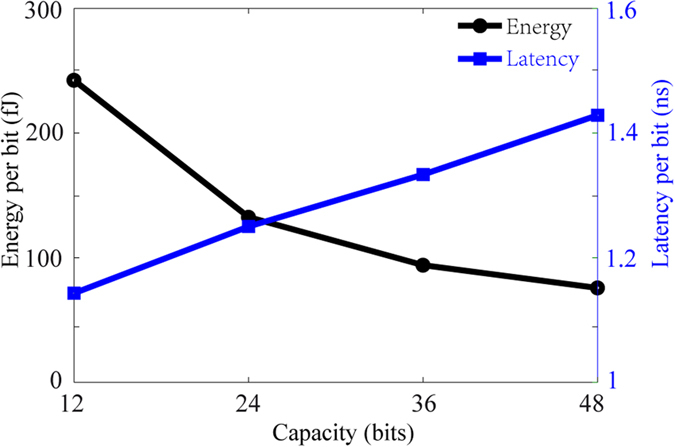
The dependence of the energy consumption and latency per bit versus RM device capacity. A 400 nm-long magnetic domain is regarded as a bit. The voltage supply is fixed to 0.53 V. A tradeoff relationship is found: higher device capacity achieves lower energy but higher latency.

Finally, we analyze the dependence of device capacity on the energy consumption and performance. The energy dissipation per bit transfer can be described as


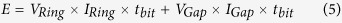


where *V*_*Ring*_ and *V*_*Gap*_ are the voltage supplies charging on the ring and gap parts, which are always the same, *I*_*Ring*_ and *I*_*Gap*_ are the currents flowing through these two parts, which depend on the material resistivity and sectional area, *t*_*bit*_ is the latency for transferring a bit, which depends on the current density. By fixing the voltage at 0.53 V and minimal adjacent DW distance at 400 nm, Figure 6 shows a tradeoff relationship among the storage capacity, speed and energy: more bits per device will degrade the speed performance and economize the energy. For example, a 24-bit ring-shaped RM can achieve 130 fJ/bit and 800 MHz. It is also shown that one third of the energy consumption can be saved with the increase of the device capacity from 12 bits to 48 bits, nevertheless the speed lightly deceases, which is in line with the expectation of the capacity improvement.

## Conclusions

In this work we proposed a ring-shaped RM, which is based on a combinative effect of DMI stabilized SOT driven chiral DW motions and STT driven DW motions. Compared to conventional stripe-shaped structure, this ring-shaped structure can remain all the data during the shifting process, without increasing overhead and requiring additional manipulations. We have validated its functionality and analyzed its performance through micromagnetic simulations. Different critical conditions for its stability have also been addressed. Thanks to its various potential advantages due to the “end to end” circulation of storage data, in terms of area, energy, geometry flexibility as well as reliability, this ring-shaped RM will vitalize the development and practical application of RM^34^,^35^.

## Methods

### Micromagnetic simulation

The micromagnetic simulations are carried out by using the open source software package MuMax3 available at http://mumax.github.io/. The three-dimensional time-dependent dynamics of DW motions is described by the modified Landau-Lifshitz-Gilbert (LLG) equation including the SOT terms (SHE and Rashba effect) shown as


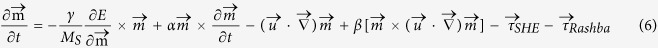


where 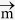 is the unit vector of local magnetization, *α* is the damping parameter, *γ* is the gyromagnetic ratio, *E* is the micro magnetic energy density. Interface DMI term is included in micromagnetic energy expression^15^,


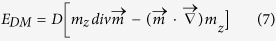


where *D* is the DMI constant.

SHE and Rashba effect are involved through the torques as follows.


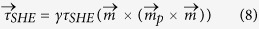



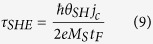


where 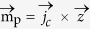, 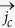 and 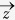 are unit vectors in the charge current and out of plane directions.


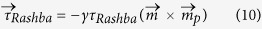



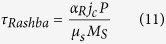


where *a*_*R*_ is the Rashba parameter.

STT effect is represented by the third and fourth terms representing the adiabatic and non-adiabatic torque respectively with u given by


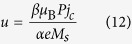


We adopt the magnetic material parameters: the Gilbert damping constant is 0.015, the non-adiabatic constant is 0.2, the spin hall angle is 0.01, the Exchange stiffness is 20 pJ/m^2^, the spin polarization rate is 0.5, the saturation magnetization is 1 MA/m, the uniaxial anisotropy is 800 kJ/m^3^, the ferromagnetic layer thickness is 1 nm, the DW width is 10 nm, the DMI parameter is 1.5 mJ/m^2^.

## Additional Information

**How to cite this article**: Zhang, Y. *et al.* Ring-shaped Racetrack memory based on spin orbit torque driven chiral domain wall motions. *Sci. Rep.* **6**, 35062; doi: 10.1038/srep35062 (2016).

## Supplementary Material

Supplementary Information

Supplementary Movie 1

Supplementary Movie 2

Supplementary Movie 3

Supplementary Movie 4

Supplementary Movie 5
