# Supplementary material for: Ring-shaped Racetrack memory based on spin orbit torque driven chiral domain wall motions
Source: Sci Rep. 2016 Oct 11;6:35062. doi: 10.1038/srep35062 (PMC5057157; doi:10.1038/srep35062)
Supplement: Supplementary Information [file srep35062-s1.doc]

**Ring-shaped Racetrack memory based on spin orbit torque driven chiral domain wall motions**

Yue Zhang^1,2,*^, Xueying Zhang^1,2,3,*^, Jingtong Hu^4^, Jiang Nan^1,2^, Zhenyi Zheng^1,2^, Zhizhong Zhang^1,2^, Youguang Zhang^1,2^, Nicolas Vernier^3^, Dafine Ravelosona^3^, and Weisheng Zhao^1,2,3,#^

*1 Fert Beijing Institute, Beihang University, Beijing, China*

*2 School of Electronic and Information Engineering, Beihang University, Beijing, China*

*3 Institut d’Electronique Fondamentale (IEF), Univ. Paris-Sud, CNRS, Orsay, France*

*4 School of Electrical and Computer Engineering, Oklahoma State University, U.S.A.*

*These authors contributed equally to this work.

#E-mail: weisheng.zhao@buaa.edu.cn

**SUPPLEMENTARY INFORMATION**

**Dependence of different configuration DW motions on racetrack width**

As shown in Fig. S1, we tried five widths, i.e. 40 nm, 80 nm, 120 nm, 160 nm and 200 nm, keeping the diameter of the ring 512 nm. We found that the velocities of different DW configurations (up-down and down-up) had not an obvious difference (also see Supplementary Movie 4).


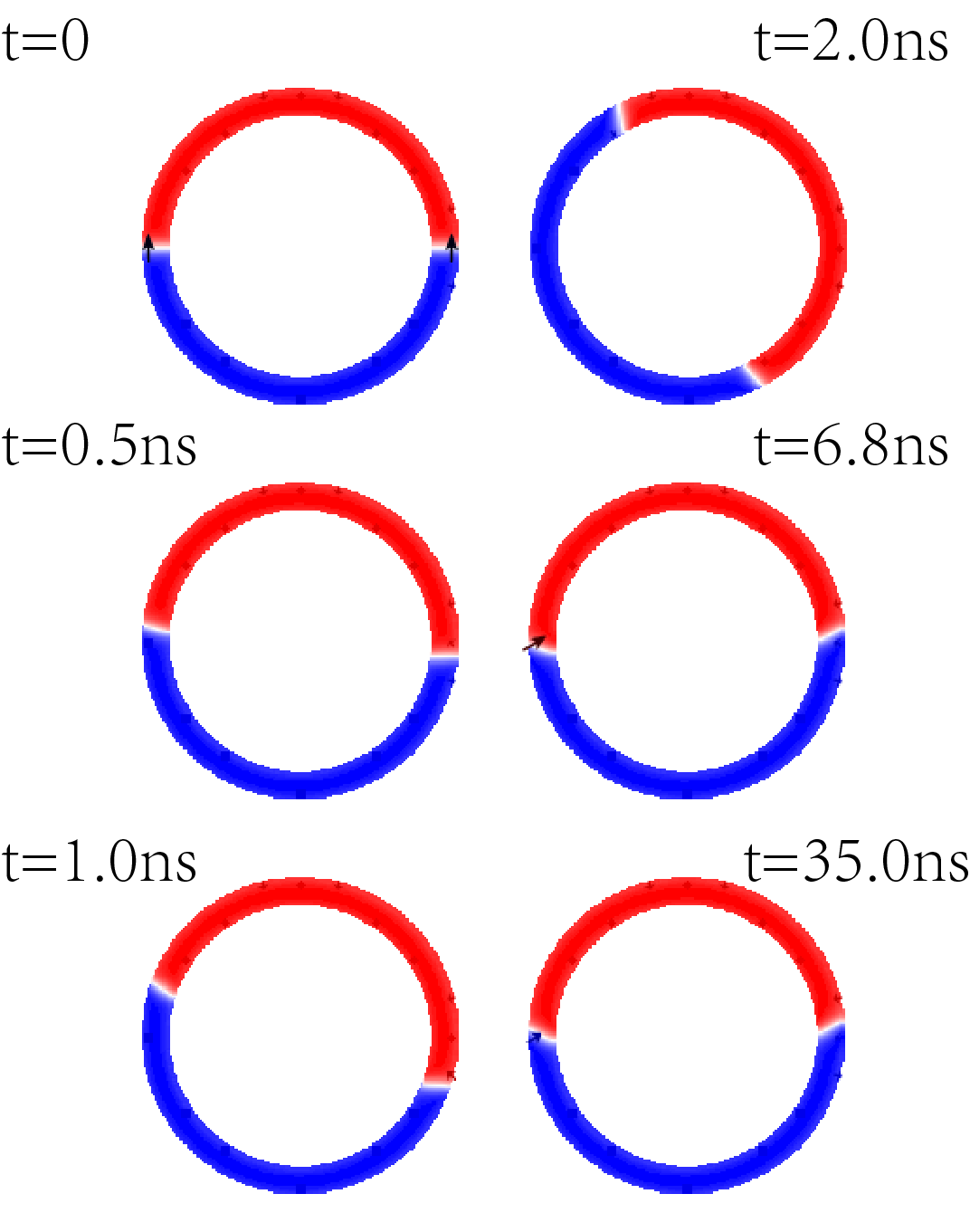

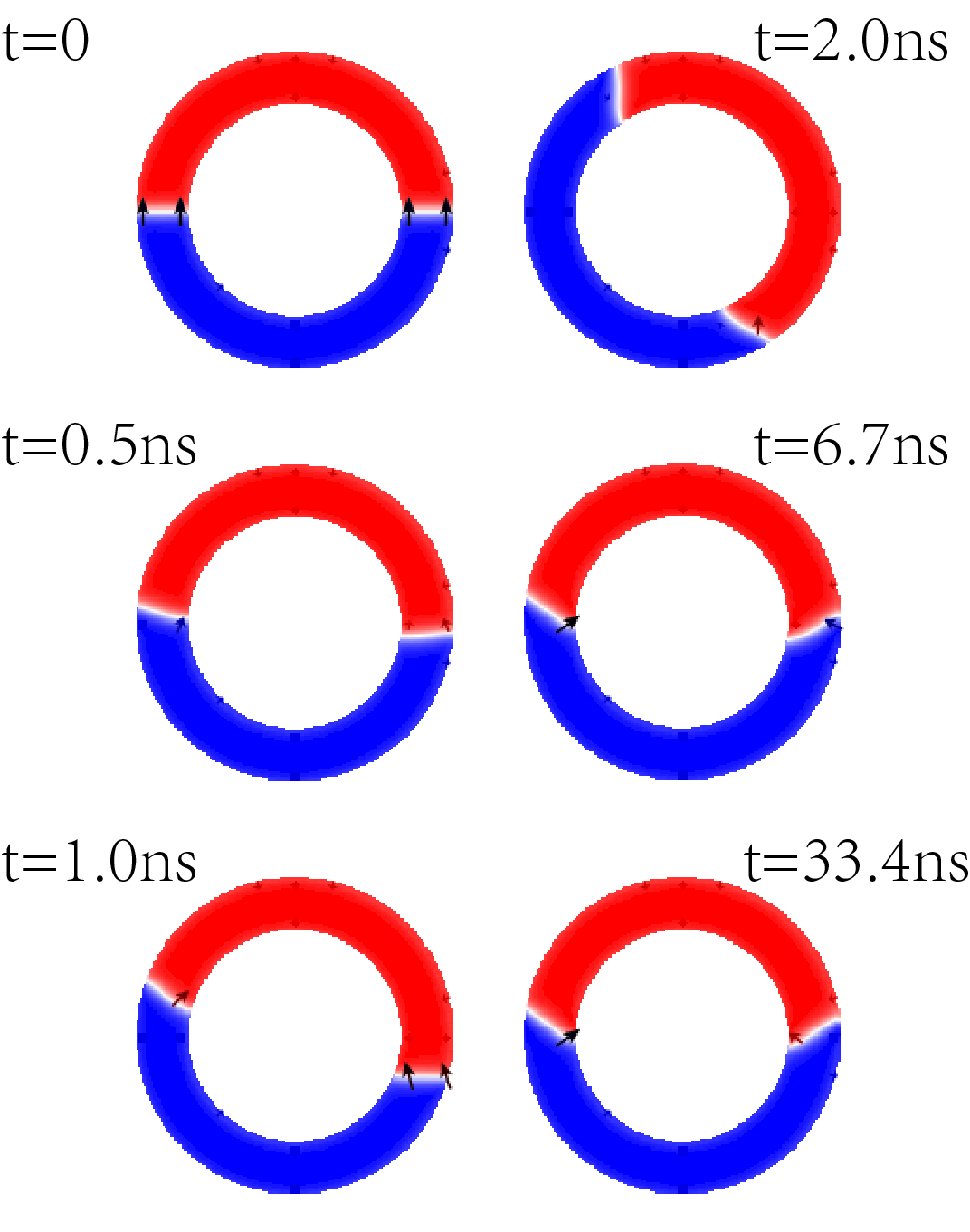

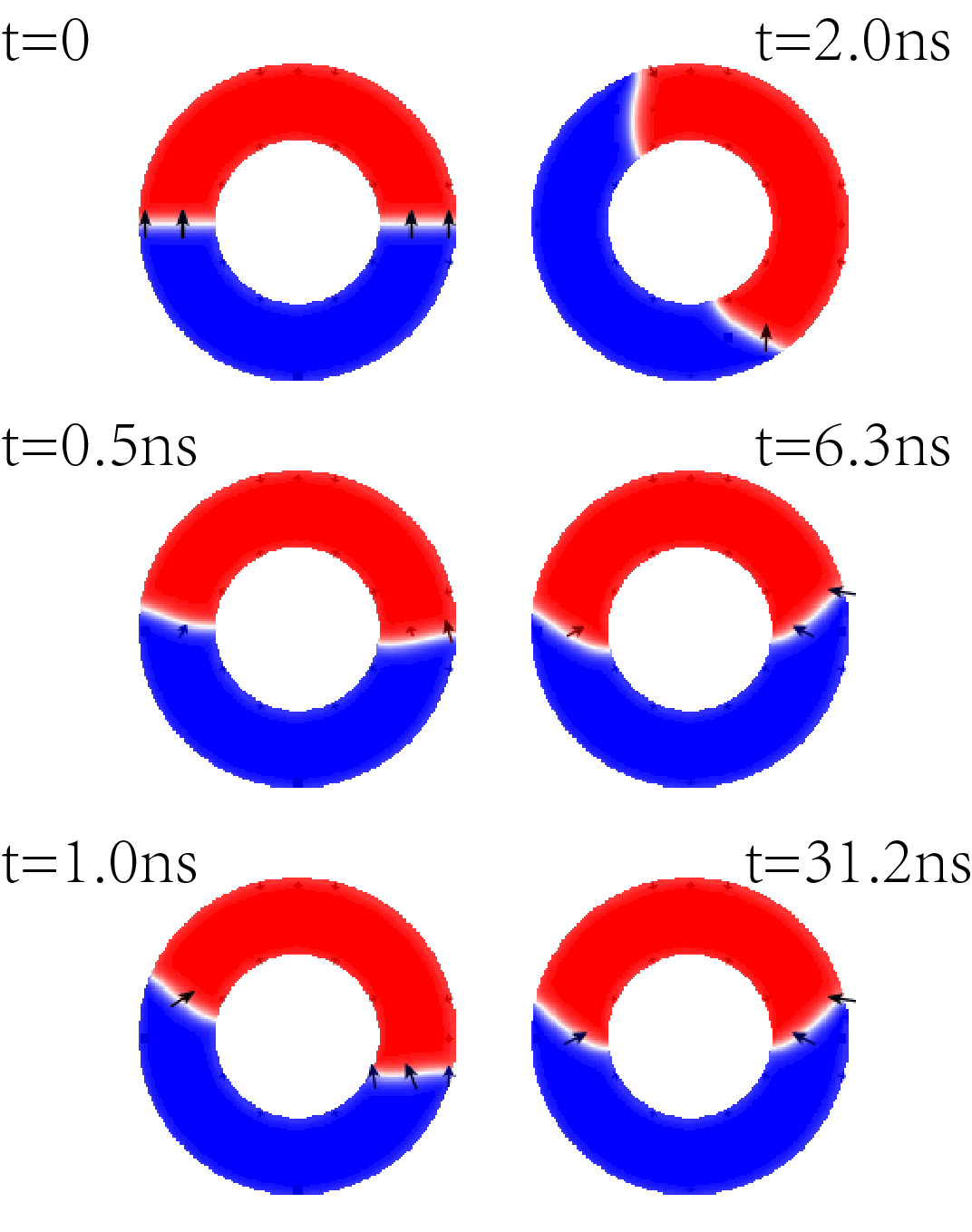


(a) (b) (c)


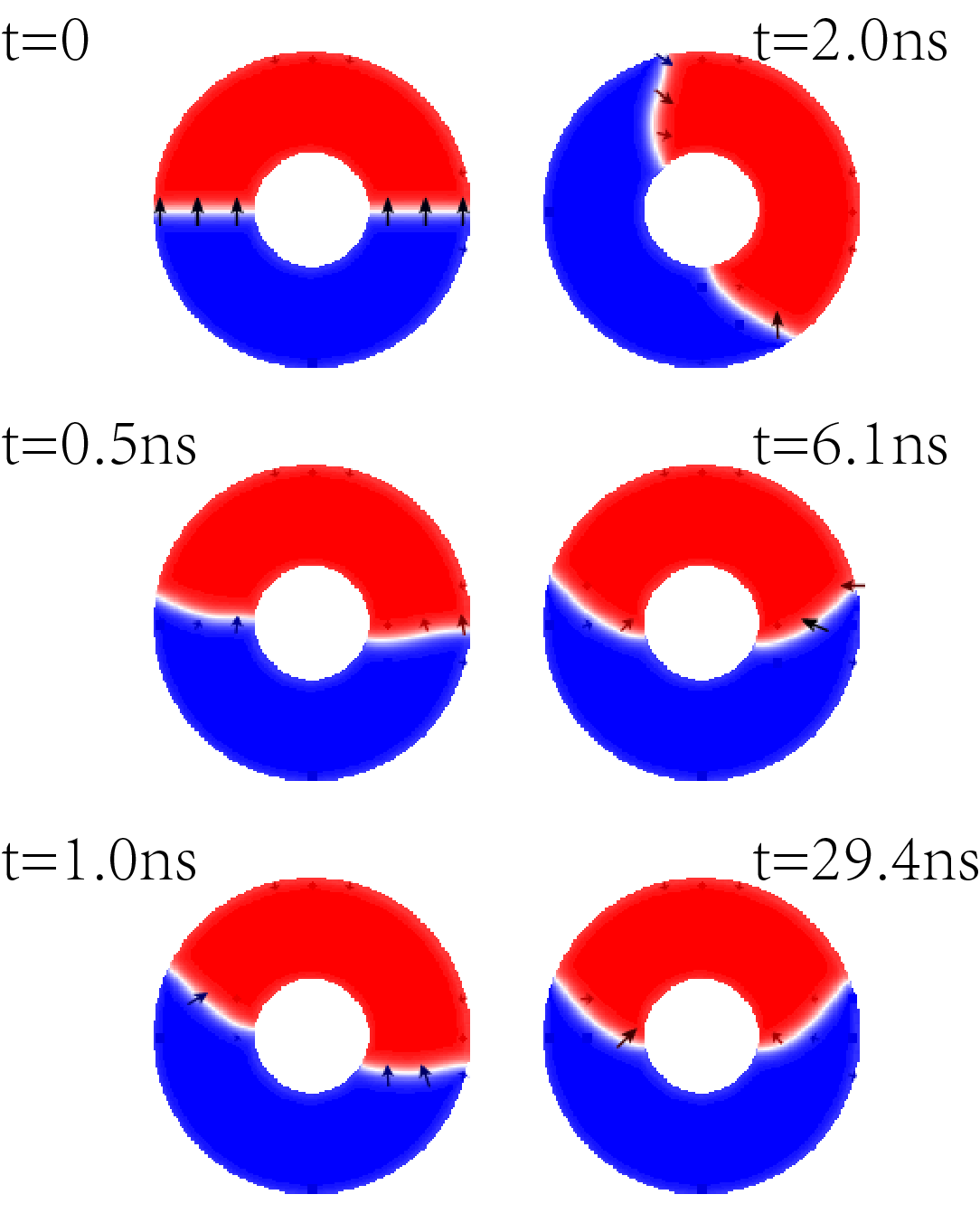

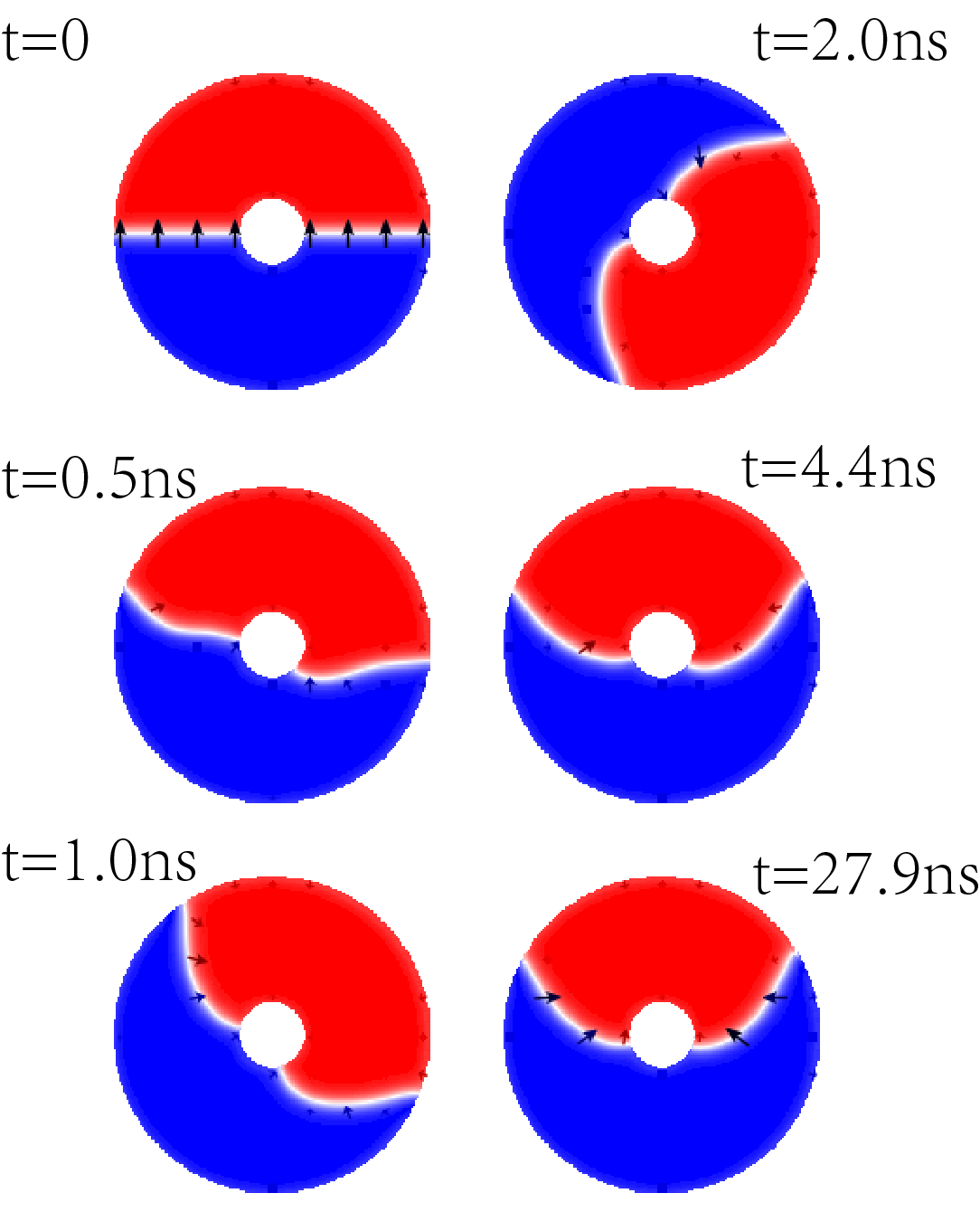


(d) (e)

Fig. S1. Dependence of different configuration DW motions on racetrack width. Diameter is 512 nm. (a) Racetrack width is 40 nm. (b) Racetrack width is 80 nm. (c) Racetrack width is 120 nm. (d) Racetrack width is 160 nm. (e) Racetrack width is 200 nm.

From the results, we analyzed the ring-shaped structure from the theoretical point of view. As shown in Fig. S2, the polarization of the induced spin current is always along the radial direction, since the charge current is flowing along the ring. This is always symmetrical as the case of the DW motions in the stripe. In addition, the curvature sign of ring could lead the tilting angle change with respect to different DW configurations. However, as mentioned in the paper, this change will be at a cost of a higher DW energy. In other words, this change will be minimized as much as possible. The related theory has been confirmed by *O. Boulle, et al. “Domain wall tilting in the presence of the Dzyaloshinskii-Moriya interaction in out-of-plane magnetized magnetic nanotracks”, Phys. Rev. Lett., 111, 217203, 2013*. Although they discussed the stripe-shaped case, the viewpoint of energy compensation can be referred. Therefore, we didn’t find the velocity difference by altering the racetrack width.


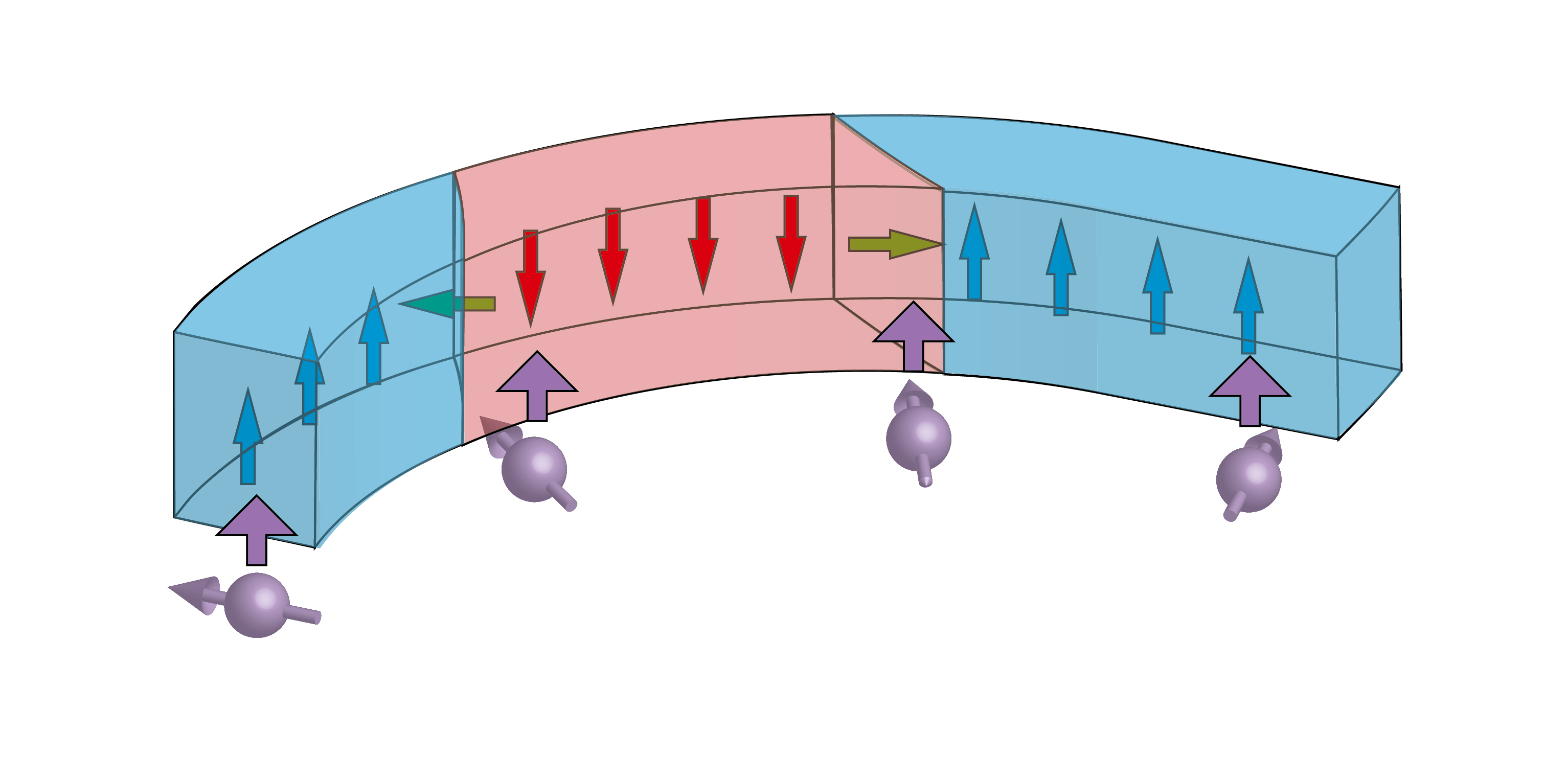


Fig. S2. Magnetization dynamics driven by SOT in curved trajectory.

**Impact of pinning notches on the ring-shaped RM**

We studied in details the impact of tiny notches at the border of the ring, which could generate a random pinning effect for the DW motions (see Fig. S3). As the notches are randomly set on the racetrack, its impact can also be referred for the other pinning factors, such as inhomogeneous exchange stiffness or anisotropy. From the simulation results, the DW motion velocity is influenced by the pinning notches. However, the difference is relatively low. By decreasing the size of notches from 4 nm to 1 nm, the velocity difference between different DW configurations becomes even smaller (see Tab. S1). The positive or negative difference demonstrates the randomness. The smallest difference can be as low as 0.1%, which can be neglected for the real application. This analysis shows that we can decrease the velocity difference by improving the technical process for minimizing the notch size. Moreover, we studied the dependence of velocity difference on spin current density (see Fig. S4). We found that the higher current density could decrease the velocity difference, as the velocity is saturated with a high current density and the impact of pinning notches is mitigated.


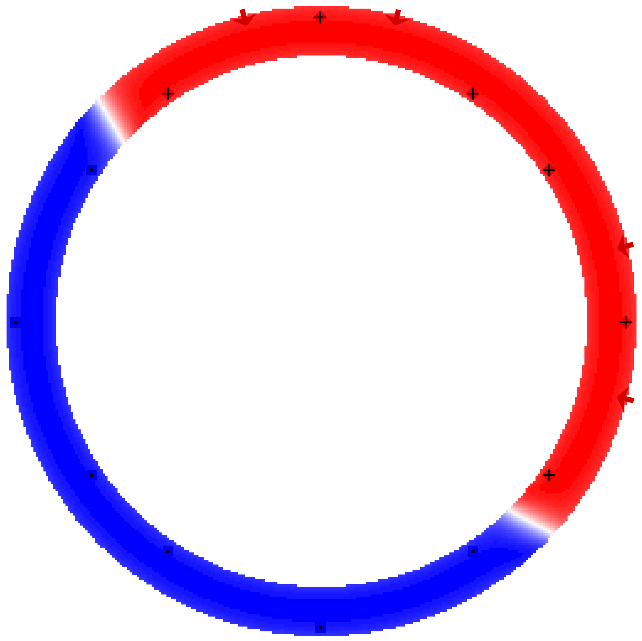

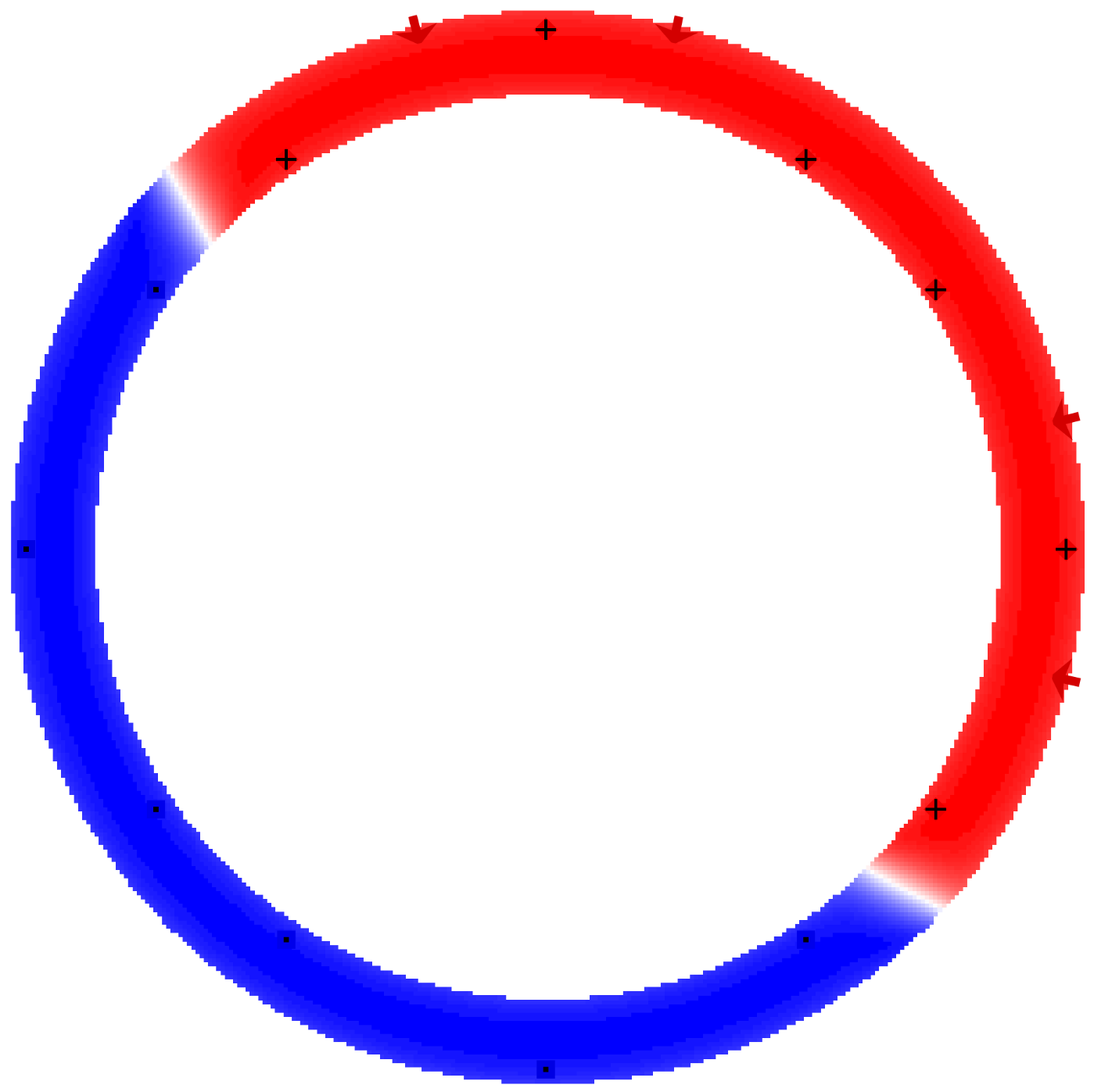


Fig. S3. Tiny notches randomly set along the ring-shaped racetrack.

Table. R1. Dependence of velocity difference on size of pinning notches

| Notch size (nm) | 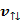 (m/s) | 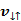 (m/s) | Average velocity (m/s) | Velocity difference |
| --- | --- | --- | --- | --- |
| 1 | 256.30 | 256.57 | 256.43 | -0.1% |
| 2 | 254.99 | 255.36 | 255.17 | -0.14% |
| 2.5 | 248.79 | 248.00 | 248.39 | 0. 32% |
| 3 | 244.88 | 244.32 | 244.60 | 0. 23% |
| 4 | 210.34 | 199.19 | 204.76 | 5.44% |

Fig. S4. Dependence of velocity difference on spin current density.

**Adding artificial pinning sites for unifying different DW motions**

Micromagnetic simulations have been performed to demonstrate the effect of pinning sites on the reliability of the ring-shaped RM. From the results, by applying current pulses for driving DW motions, the pinning sites can halt the DWs at the artificial positions. In this case, the RM is stable and reliable after numbers of times of data circulations (See Supplementary Movie 5).

**Impact of non-uniform current distribution along the radical direction**

Firstly, the uniform current distribution in conductor loop is widely acknowledged. In our proposed RM, the current is injected in the heavy metal wire, which can be considered as a conductor loop. If the metal wire has a high conductivity, the average length is much larger than its thickness and its cross-sectional area is constant, the current distribution within the ring is essentially uniform. This has been confirmed by certain literatures, for example, R. M. Fano, et al., “Electromagnetic fields, energy, and forces”, 1960, John Wiley & Sons.

In our study, we use Pt as the heavy metal wire which has a high conductivity, the device radius is 1.024 µm which is much larger than the thickness (2 nm), and the cross-sectional area is constant. All of these conditions can confirm the uniform current distribution in our nanometer-size device.

In addition, we still study the impact of non-uniform current distribution on our proposed RM by highlighting this non-uniformity. For simplicity, we assume that the current reduces along the radical direction in proportion to the radius. The average current density is 3x10^11^ A/m^2^. As shown in Fig. S5, the non-uniform current distribution influences the DW angle. However, if the length is much larger than the width, the impact is limited and the DW motions keep stable. If the width increases, the impact will be more significant and lead to certain unexpected issue. These phenomena can be explained as follows: when the width is relatively small, the current difference in the ring is limited. Although the DW angle changes, the increased DW energy by enlarging the DW surface will compensate this angle change and stabilize DW motions. If the width is too large, the current difference along radical direction will greatly increase, the balance between DW angle and DW motions will thus be broken.


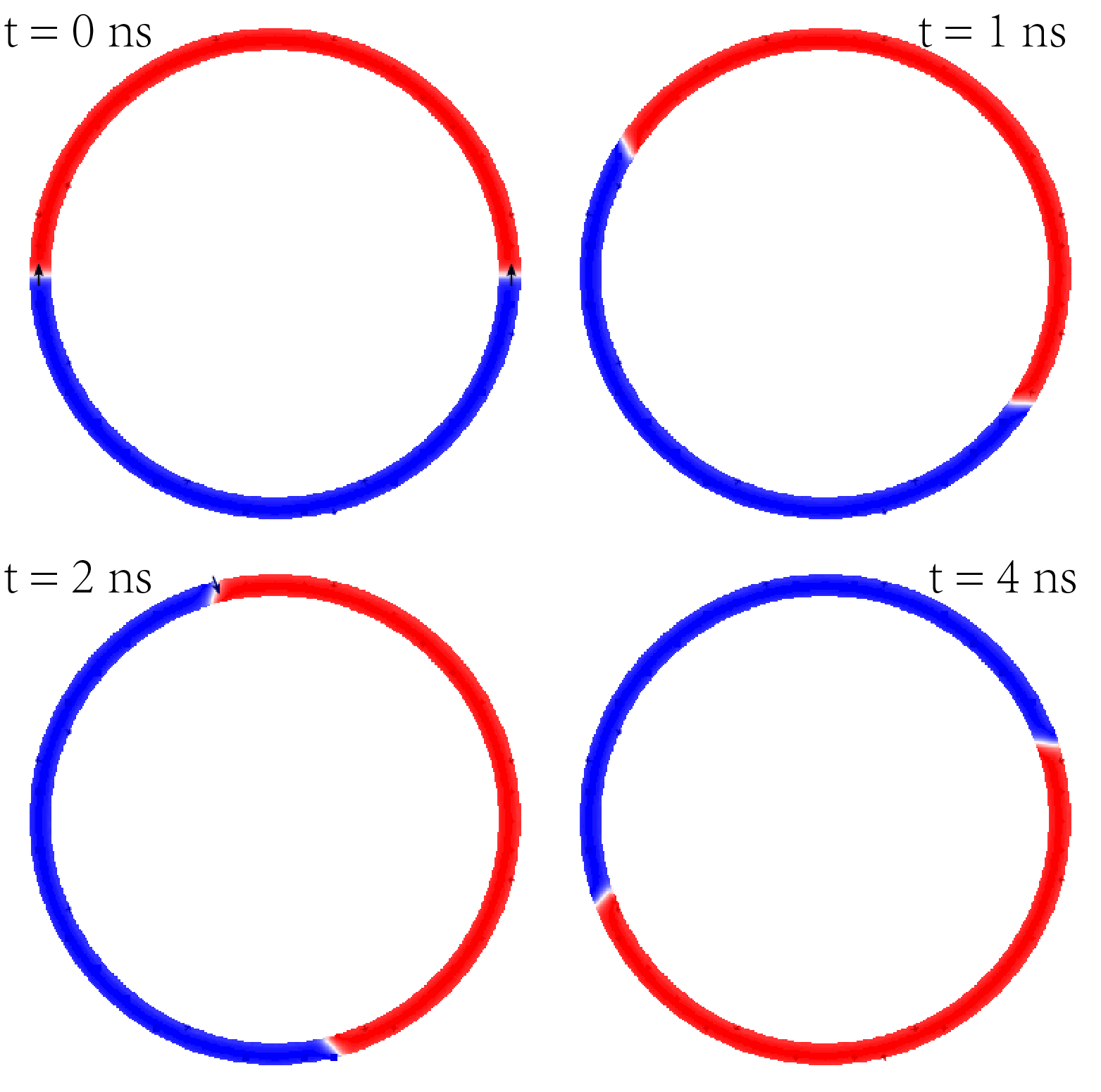

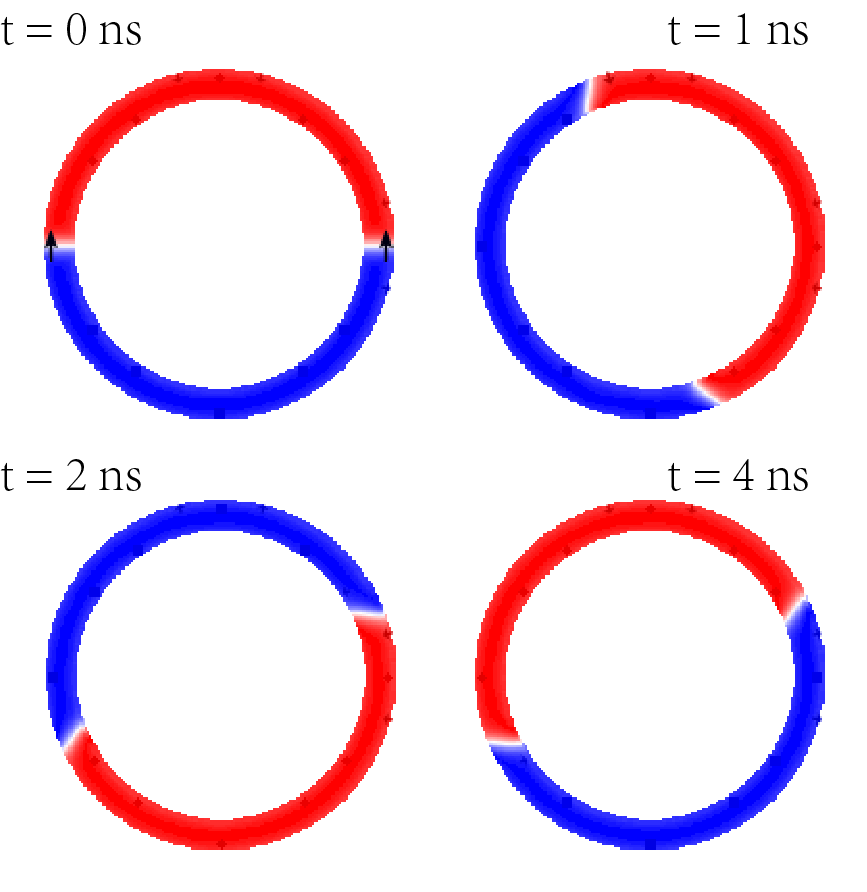


(a) (b)


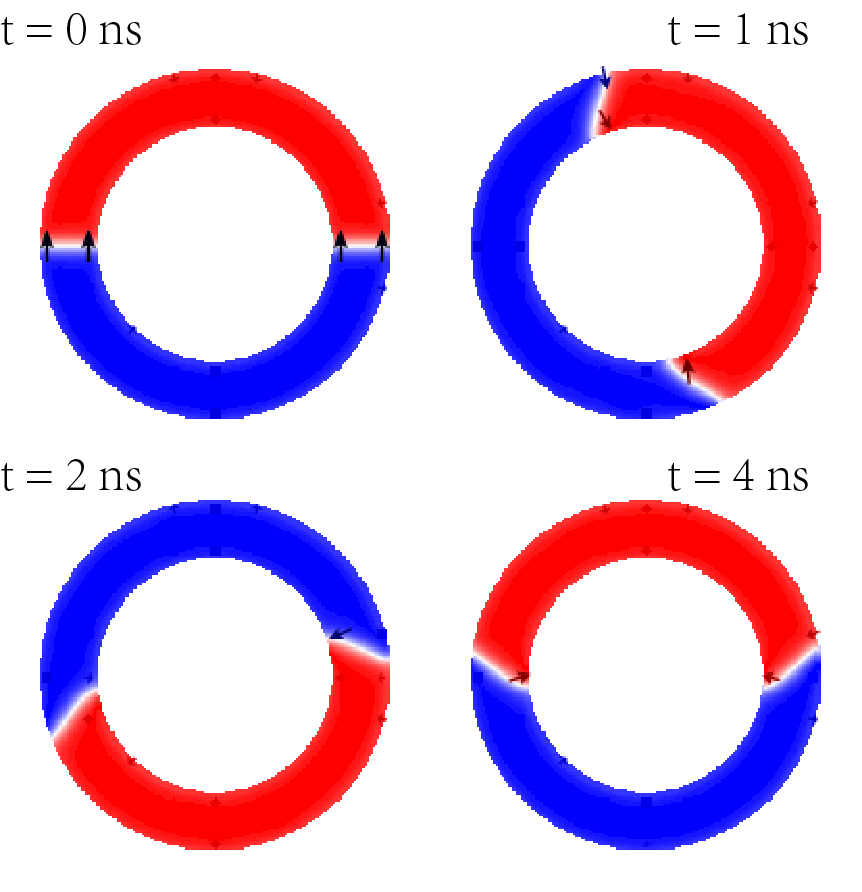

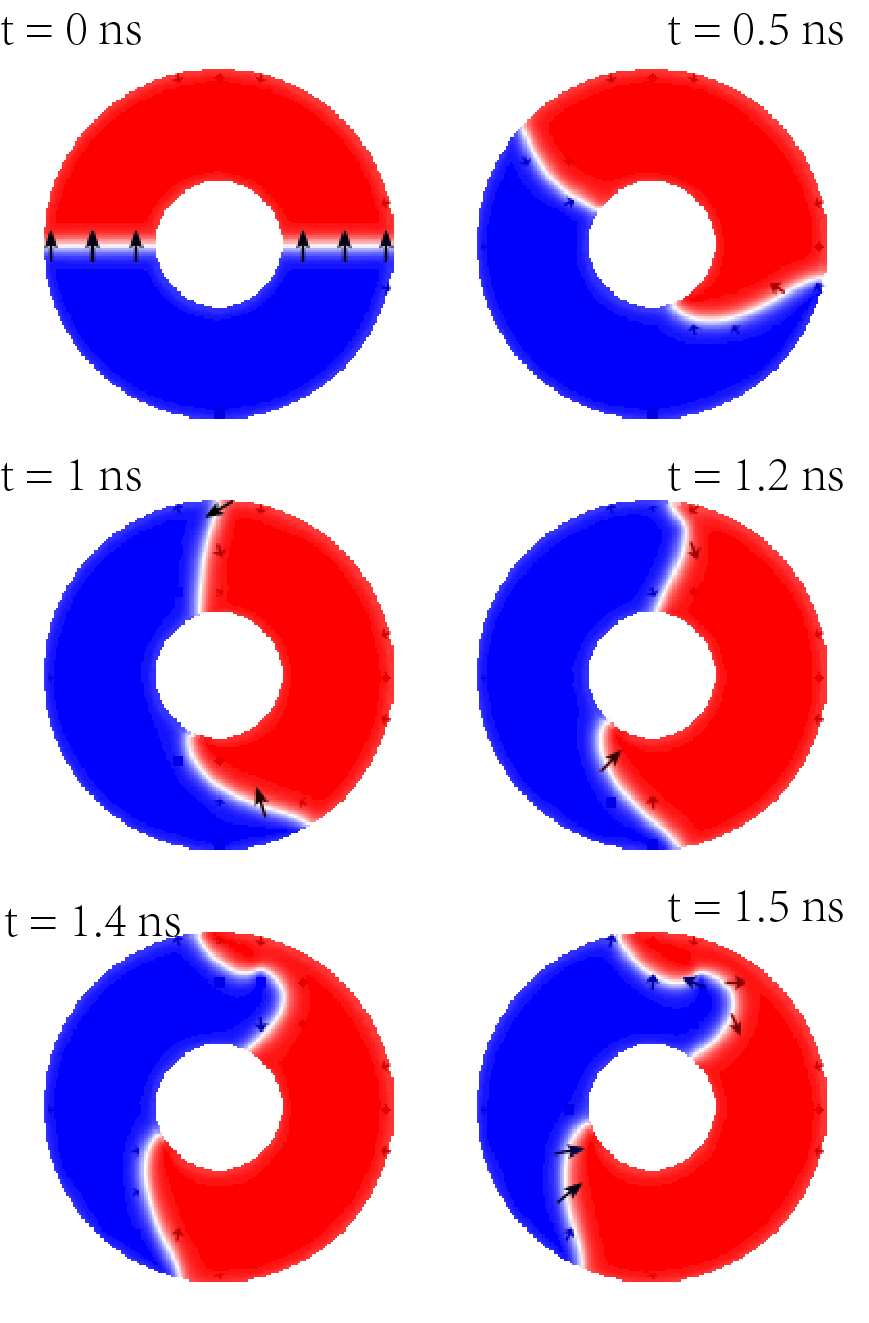


(c) (d)

Fig. S5 Impact of non-uniform current distribution on ring-shaped RM for various widths. (a) Width is 40 nm and diameter is 1024 nm. (b) Width is 40 nm and diameter is 512 nm. (c) Width is 80 nm and diameter is 512 nm. (d) Width is 160 nm and diameter is 512 nm.

In summary, the current distribution should essentially be uniform in our proposed RM, in which the conductivity of heavy metal is high, the length is much larger than the thickness and the cross-sectional area is constant. Only if we focus on the non-uniform current distribution in ring-shaped RM with a large nanowire width, the device functionality will be influenced. However, in order to improve the storage capacity and density, the ring-shaped RM with a small width is the target of our work. In this case, the device functionality can be assured.

**Supplementary Movie Captions**

*In all supplementary movies, the red regions represent +z magnetization, blue ones represent –z magnetization.*

**Supplementary Movie 1.** SOT driven chiral DW motions in the “Straight” (Pt(2nm)/CoFeB(0.6nm)/MgO), whose width is 40 nm and length is 1.28 um. The velocity can reach 400 m/s with a current of $5\times10^{11}$ A/m^2^, corresponding to Figure 2a-d of the main text.

**Supplementary Movie 2.** SOT driven chiral DW motions in the “Bend” (Pt(2nm)/CoFeB(0.6nm)/MgO), whose external diameter is 1.024 um and width is 40 nm. The gap length is 100 nm. Current injected into the heavy metal layer *J_Ring_* is $3\times10^{11}$ A/m^2^ and leakage current in the gap *J_Gap_* is $1.4\times10^{12}$ A/m^2^, corresponding to Figure 2e-h of the main text.

Supplementary Movie 3. SOT driven chiral DW motions in the 16-bit ring-shaped RM. Two gaps and symmetric pinning sites are applied. *J_Gap_* and *J_Ring_* are $5\times10^{12}$ A/m^2^ and $1\times10^{12}$ A/m^2^. 0.6 ns current pulse can induce DW motions due to the inertia, corresponding to Figure 3 of the main text.

**Supplementary Movie 4.** SOT driven chiral DW motions in ring-shaped RM. Nanowire width is 40 nm, 80 nm, 120 nm, 160 nm and 200 nm, curvature diameter is 512 nm.

**Supplementary Movie 5.** SOT driven chiral DW motions in ring-shaped RM with artificial pinning sites.
