# Supplementary figures and images for: Ring-shaped Racetrack memory based on spin orbit torque driven chiral domain wall motions
Source: Sci Rep. 2016 Oct 11;6:35062. doi: 10.1038/srep35062 (PMC5057157; doi:10.1038/srep35062)

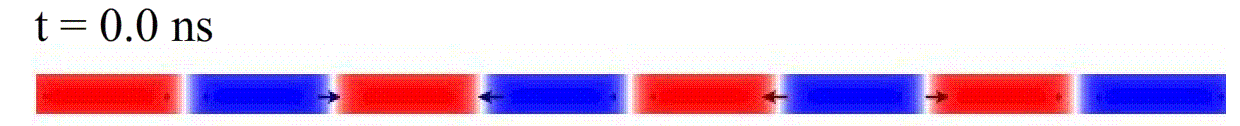

Supplement: Supplementary Movie 1 [file srep35062-s2.gif]

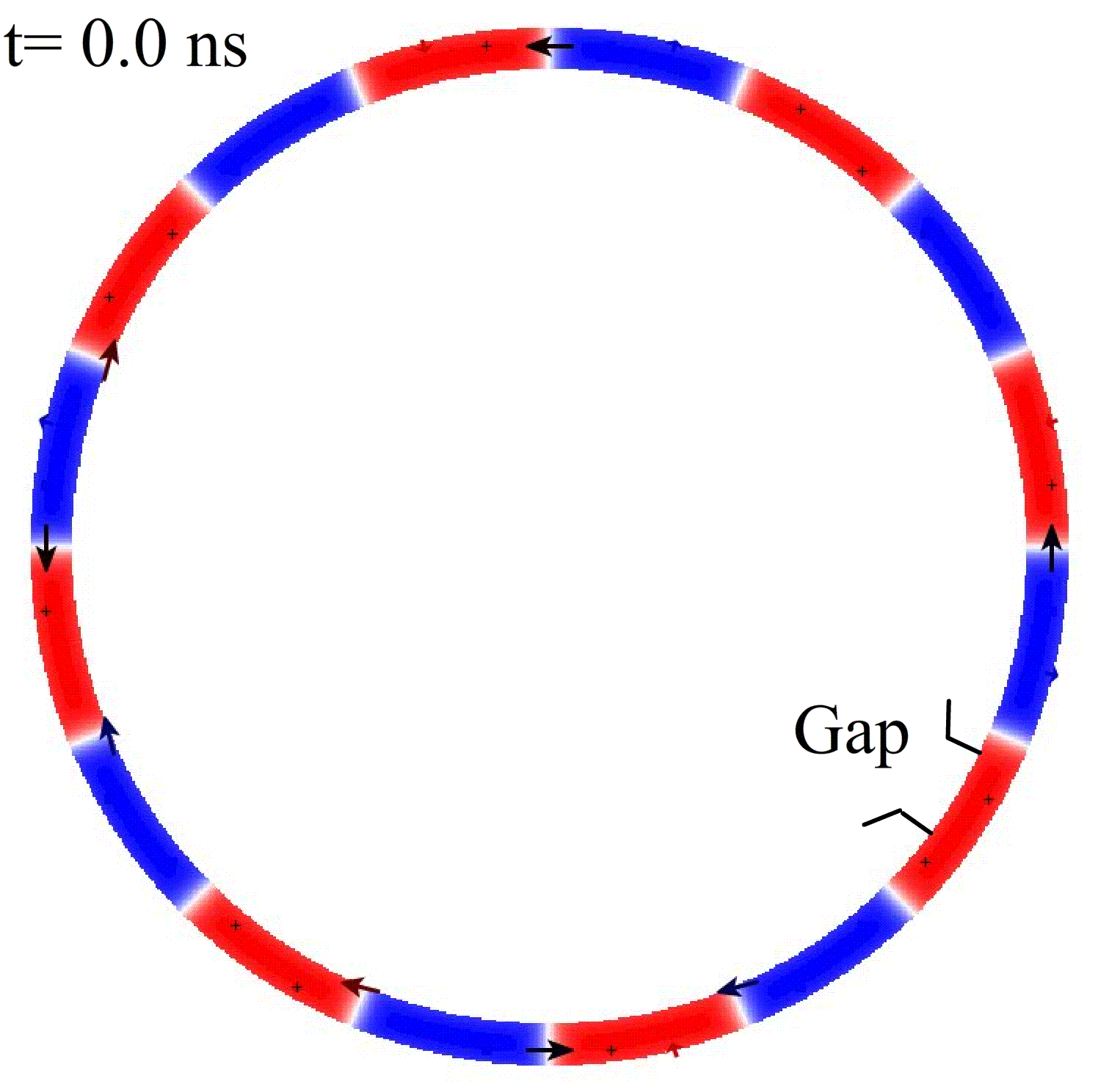

Supplement: Supplementary Movie 2 [file srep35062-s3.gif]

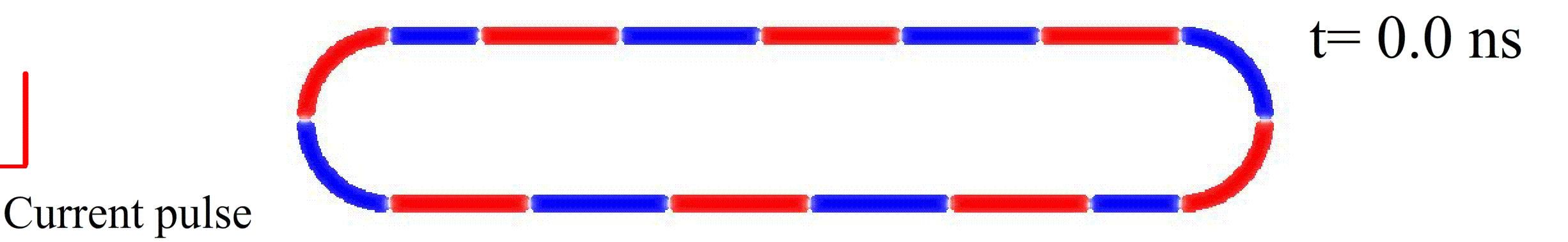

Supplement: Supplementary Movie 3 [file srep35062-s4.gif]

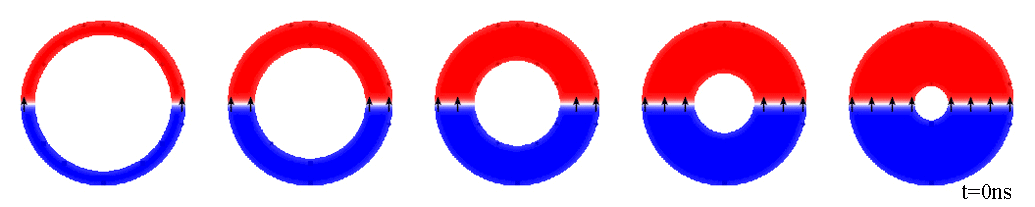

Supplement: Supplementary Movie 4 [file srep35062-s5.gif]

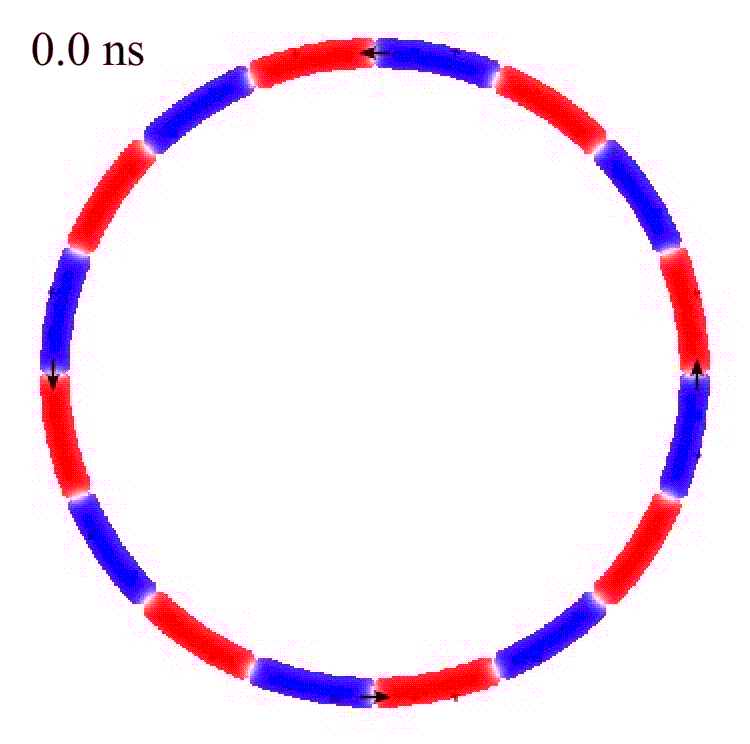

Supplement: Supplementary Movie 5 [file srep35062-s6.gif]
